# Effect of SMS Reminders, Telephone Calls, and Transport Incentives on Enhancing the Completion of Tuberculosis Diagnosis and Initiation of Treatment for Diagnosed Patients: Protocol for a Randomized Controlled Trial

**DOI:** 10.2196/70325

**Published:** 2025-11-25

**Authors:** Rebecca Nuwematsiko, Noah Kiwanuka, Lynn Atuyambe, Irene Wobusobozi, Vicent Kasiita, Samuel Kagongwe, Ronald Ssenyonga, Joan Nakayaga Kalyango, Victoria Nankabirwa, Esther Buregyeya

**Affiliations:** 1 Department of Disease Control and Environmental Health, School of Public Health, Makerere University, Kampala, Uganda; 2 Department of Epidemiology and Biostatistics, School of Public Health, Makerere University, Kampala, Uganda; 3 Department of Community Health and Behavioural Sciences, School of Public Health, Makerere University, Kampala, Uganda; 4 Infectious Diseases Institute, Makerere University, Kampala, Uganda; 5 Department of Public Health, Faculty of Health Sciences, Muni University, Arua, Uganda; 6 Clinical Epidemiology Unit, College of Health Sciences, Makerere University, Kampala, Uganda

**Keywords:** tuberculosis, TB, presumptive tuberculosis, SMS, telephone calls, transport incentives, linkage to TB care

## Abstract

**Background:**

Globally, tuberculosis (TB) programs have enhanced efforts to improve case detection, treatment initiation, and monitoring of treatment outcomes. However, less attention has been given to reducing the number of persons with presumed TB who never get tested for TB or those with confirmed TB who never start treatment in endemic regions such as Uganda. Such losses hinder progress toward attaining the 2035 End TB goals. The World Health Organization recommends mobile health (mHealth) interventions such as SMS reminders, telephone calls, mobile apps, and digital monitoring devices to foster universal health coverage. To our knowledge, there is limited evidence on whether these mHealth interventions can increase linkage to care for persons with presumed TB, particularly in sub-Saharan Africa.

**Objective:**

We aim to conduct a randomized controlled trial (MILEAGE4TB) whose aim is to assess the effect of SMS reminders, telephone calls, and transport incentives on improving completion of TB diagnosis among persons with presumed TB and initiation of treatment for diagnosed patients in Uganda.

**Methods:**

This will be a 5-arm individual randomized controlled trial among persons with presumed TB aged 18 years or older who are referred for Xpert MTB/RIF testing. Participants will be randomly assigned (2:2:2:1:1) to (1) standard of care, (2) SMS reminders only, (3) telephone calls only, (4) SMS reminders and a transport incentive, and (5) telephone calls and a transport incentive. An estimated sample size of 2389 participants will be considered. The primary outcome will be completion of TB diagnosis, defined as submitting a sputum sample for Xpert MTB/RIF testing and receiving test results within 30 days of being identified as having presumptive TB. The secondary outcomes will include (1) TB treatment initiation, which will be defined as starting TB treatment within 30 days of being diagnosed; (2) median turnaround times for TB diagnosis and treatment initiation; and (3) acceptability and feasibility of the interventions. Participants will be followed for 30 days to check whether they have tested for TB and collected their results. Chi-square tests will be performed for categorical outcomes. Analysis will be by intention to treat. Modified Poisson regression models will be used to estimate the effects of the interventions on completion of TB diagnosis and treatment initiation.

**Results:**

The study was funded in June 2020 and data collection for the trial started in August 2023. Results from this trial are not yet available. As of August 28, 2025, a total of 2355 participants had been recruited. Data from the preliminary analysis will be ready by December 2025 after all trial activities.

**Conclusions:**

This randomized controlled trial will provide insights on the use of mHealth interventions to improve the completion of TB diagnosis among persons with presumed TB and initiation of treatment for diagnosed patients.

## Introduction

### Background

Tuberculosis (TB) remains one of the leading causes of death from a single infectious agent despite the fact that it is preventable [1]. The End TB Strategy of the World Health Organization (WHO) calls for reduction of TB incidence by 90%, deaths by 95% and eliminating catastrophic costs for TB-affected households by 2035; however, progress is hindered by the loss to follow-up (LTFU) of patients along the continuum of care [2]. Patient LTFU can occur after they are identified with presumptive TB but are never tested, while those who are tested and are confirmed to have TB can also undergo LTFU and never initiate TB treatment. This is in addition to the number of patients who never access a TB diagnostic test [3]. In 2023, an estimated 10.84 million people fell ill with TB, of whom 8.16 million were diagnosed and notified to national TB programs, leaving out 2.7 million patients (25%) [1]. A study by Kim et al [4] on TB care cascades of 30 high TB burden countries found that 35% of people with TB did not receive a diagnosis, while 5% of people who were diagnosed did not initiate treatment.

The WHO recommends the use of mobile health (mHealth) for improved service delivery and continuation of health care, given its timeliness, popularity, and affordability [2,5]. SMS reminders, telephone calls, and mobile apps are some of the interventions being used to support TB care and reduce patient LTFU during the treatment phase [6-10]. These mHealth interventions have shown promise in improving adherence to TB treatment in previous studies [11-14]; therefore, they have the potential to also support persons with presumed TB to complete the diagnostic process. Incentives given to patients have also been found to improve health care outcomes elsewhere [15-19]. However, there is limited evidence on the application of mHealth interventions and incentives during the phase when one is being presumed for TB and on how they can improve completion of the diagnostic process.

Uganda’s TB burden has persisted over the years, and the country is still ranked among the 30 countries in Africa with a high burden of both TB and HIV, with an incidence of 198 cases per 100,000 population [1]. Recent studies from Uganda have reported prediagnosis LTFU of up to 40% [20] and pretreatment LTFU of 19% [21]. Diagnosis and initiation of TB treatment are critical for controlling TB transmission and achieving good patient outcomes, particularly when medication adherence is maintained [2]. Therefore, this situation calls for bold actions and innovations to reduce the number of patients lost to follow-up before completing TB diagnosis (prediagnosis LTFU) and those lost to follow-up before initiating treatment when diagnosed (pretreatment LTFU). In Uganda, mHealth interventions have emerged as promising tools to enhance TB care by improving patient outcomes and health care processes. These mHealth interventions have been used to address challenges in TB care, including contact investigation, delivery of test results, adherence to appointments, linkage to care, and treatment adherence [6,22].

The consequences of prediagnosis and pretreatment LTFU are serious. Patients with untreated TB remain infectious and can transmit TB to others, while failure to initiate treatment contributes to increased morbidity and mortality [23-26]. Therefore, monitoring the outcomes of persons with presumed TB is an important aspect of TB control [5]. This study will thus assess the effect of SMS reminders, telephone calls, and transport incentives on enhancing completion of TB diagnosis and initiation of treatment in Uganda. Assessing the effect of these interventions will provide evidence on whether reminding patients using SMS reminders or telephone calls, with or without conditional cash incentives, can enhance completion of TB diagnosis and initiation of treatment for improved patient outcomes and hence aid in designing targeted interventions and policies.

### Goal of the Study

This randomized controlled trial aims to assess the effect of SMS reminders, telephone calls, and transport incentives sent to persons with presumed TB and those with confirmed TB on enhancing completion of TB diagnosis and initiation of treatment for diagnosed patients. The TB care continuum comprises 4 steps: screening, diagnosis, treatment initiation, and treatment completion [3,4]. This study will address LTFU along the TB care continuum at 2 time points: before diagnosis and before treatment*.* Prediagnosis LTFU refers to the failure to test for TB and obtain results within 30 days from the date of being presumed. Pretreatment LTFU refers to failure to initiate treatment after TB has been confirmed. The proposed intervention will use SMS reminders and telephone calls to improve communication between patients and health care providers, while the transport incentives will help address the economic barriers to diagnosis completion and treatment initiation.

### Objectives and Hypothesis

The primary objective is to assess the effect of SMS reminders, telephone calls, and transport incentives on completion of TB diagnosis among persons with presumed TB and initiation of treatment for diagnosed patients. The secondary objectives are to determine the median turnaround time for TB diagnosis and treatment initiation and to examine the acceptability and feasibility of the interventions. We hypothesize that compared to standard of care (SOC), each individual intervention—SMS reminders alone or telephone calls alone—will improve completion of TB diagnosis and initiation of treatment by reducing prediagnosis LTFU by a minimum of 40% (from 24% to 14.4%), while a combined intervention—SMS reminders or telephone calls coupled with transport incentives—will reduce prediagnosis LTFU by 50% (from 24% to 12%). This protocol follows the SPIRIT (Standard Protocol Items: Recommendations for Interventional Trials) reporting guidelines [27].

## Methods

### Study Setting

The study will be conducted in 6 government health facilities in central Uganda: Kiruddu National Referral Hospital, Entebbe Regional Referral Hospital, Mukono General Hospital, Luwero General Hospital, Kawolo General Hospital, and Wakiso Health Center (HC) IV (a lower-level facility). The selection of these health facilities ensures a mix of urban and rural settings and different administrative levels to increase the generalizability of the findings. Uganda’s government health system consists of national referral hospitals, regional referral hospitals, and the district health system [28]. The district health system is further divided into district general hospitals; HCs IV, III, and II; and village health teams [28,29]. National referral hospitals provide comprehensive specialist services, while regional referral hospitals have specialists in only some specific fields. District general hospitals and HC IVs have physicians. HCs are graded based on the administrative zone served, population size, and services offered. HC IVs serve a county or municipality with a population of approximately 100,000 people and provide emergency surgery, blood transfusion, and all general medical services offered at lower-level health facilities. HC IIIs serve a subcounty with a population of approximately 20,000 people and provide maternity, inpatient, and laboratory services in addition to all health services offered at an HC II. HC IIs provide health services to a parish, ward, or division with a population of approximately 5000 people and offer outpatient care, prenatal care, immunization, and community outreach services [29].

**Figure 1.**
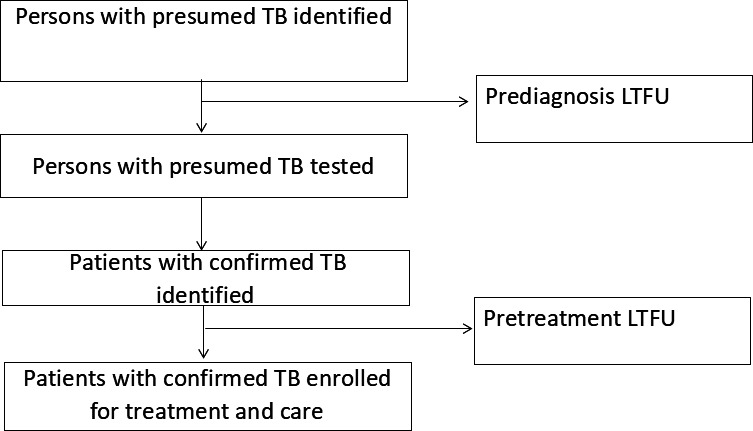
Patient flow through the study facilities. LTFU: loss to follow-up; TB: tuberculosis.

In the study facilities, patients presenting with symptoms suggestive of TB (eg, cough, fever, weight loss, and night sweats) are identified and their demographic, contact, and clinical details recorded in the registers for persons with presumed TB at the different facility entry points. Patients are evaluated for TB using Xpert MTB/RIF, urine lipoarabinomannan, or chest X-ray depending on their symptoms, with diagnosis primarily conducted via Xpert MTB/RIF as recommended by the Ministry of Health [30]. Persons with presumed TB are offered sputum testing using Xpert MTB/RIF or sputum smear microscopy, while those unable to produce sputum are offered alternative diagnostic tests (eg, chest X-ray, urine lipoarabinomannan, sonography, tissue biopsy, and cerebrospinal fluid analysis). Details of patients who complete the required investigations are subsequently recorded in the laboratory or radiology registers. Thereafter, patients confirmed to have TB are started on treatment and recorded in the unit TB register (Figure 1). Ideally, the results of Xpert MTB/RIF tests should be available within 2 hours, allowing persons with presumed TB to receive their results on the same day as sputum submission. However, challenges such as high patient loads, cartridge shortages, lack of on-site Xpert MTB/RIF equipment, and low staffing levels in laboratories, among others, often prevent same-day completion of the diagnostic process, contributing to a considerable proportion of patients being lost to follow-up [31-33].

### Trial Design, Population, and Eligibility

This will be a multicenter individual randomized controlled trial with five arms: (1) SOC (control arm), (2) SMS reminders only, (3) telephone calls only, (4) SMS reminders and a transport incentive, and (5) telephone calls and a transport incentive. This intervention was designed and adapted through stakeholder engagements in a co-design workshop. The original design included SMS reminders only and a transport incentive [34]. However, based on stakeholder engagements, telephone calls were added to address the following concerns: (1) the risk of unintentional disclosure if someone other than the recipient reads the SMS text message; (2) existing SMS fatigue due to high volume of messages from telecom companies, leading to a likelihood of messages being ignored or deleted; (3) lack of real-time 2-way communication; and (4) lack of usefulness for patients who cannot read. Telephone calls were preferred because they offer privacy and foster 2-way communication in real time .

### Description of the Study Arms

#### SOC Arm (Control)

Patients in the SOC arm (control) will continue to receive routine TB care, according to the Uganda Ministry of Health guidelines, including (1) screening individuals for TB, (2) identifying persons with presumed TB and recording their details in the TB register, (3) referring for testing, (4) receiving results, (5) irregular automated SMS text notification from the Xpert MTB/RIF machine to attending clinicians when results from the tests are ready, (6) irregular telephone calls to patients with positive TB test results to return to the health facility and start treatment, and (7) initiating treatment for patients with confirmed TB. There are irregularities in sending of SMS and making telephone calls due to inconsistencies in availability of funds, breakdown in the Xpert MTB/RIF server system, and limited human resource to support these processes.

**Intervention Arms**

The intervention will comprise 3 enhanced approaches to motivate participants to complete TB diagnosis: SMS reminders, telephone calls, and transport incentives. These interventions will be delivered in four arms: (1) SMS reminders only, (2) telephone calls only, (3) SMS reminders and a transport incentive, and (4) telephone calls and (5) a transport incentive.

#### SMS Reminders Only

Participants in this arm will receive up to 3 SMS reminders. The first reminder will be sent on day 0, the day of study enrollment. The second reminder will be sent once the patient’s results are ready. If the patient does not return to complete the TB diagnostic process (ie, testing and receiving results) within 1 day of the second reminder, a third reminder will be sent. This third reminder will serve as a reinforcement, reminding participants to return and complete diagnosis or initiate treatment if they have not already done so. Standardized SMS reminders will be manually sent by the research team through the telecom service providers MTN and Airtel in either Luganda (the local language) or English, based on the participant’s language preference determined at enrollment. The SMS reminders are planned to be 1-way (unidirectional) and the content neutral, excluding any mention of the word “TB” or the patient’s name. The specific wording of the SMS reminders will be co-designed with stakeholders including persons with presumed TB and health workers. Quality control officers will verify the SMS reminders sent daily, checking for the correctness of recipient telephone numbers and ensuring that all reminders are sent on time as per the study procedures. Each day, quality control officers will randomly select at least 4 participants from the SMS reminder arm or from among those scheduled to receive the second or third reminder and cross-check their details on the study telephone to confirm that the participant received the appropriate SMS reminder and in the preferred language. They will also cross-check the correctness of the telephone number that received the SMS reminder vis-à-vis the one recorded in the register for persons with presumed TB. Successful transmission of reminders will be checked by requesting sample statements from the telecom companies to confirm SMS dispatch to patients. Moreover, at follow-up, participants will be asked whether they received either an SMS reminder or a telephone call and a transport incentive, further corroborating whether the participant received the SMS reminder.

#### Telephone Calls Only

In this arm, participants will receive up to 3 telephone calls as reminders, in addition to the usual SOC. The first telephone call will be made on the day of enrollment (day 0). The second telephone call will be made once the patient’s results are ready. If the patient does not return to complete the TB diagnostic process within 1 day of the second call, a third telephone call will be made. The third telephone call will serve as a reinforcement, reminding participants to return and complete diagnosis or initiate treatment if they have not already done so. Telephone calls will be made in either Luganda (the local language) or English, based on the participant’s language preference determined at enrollment.

Daily quality checks will be carried out to ensure delivery of the intervention to participants. At least 4 participants from the telephone call arm or from among those scheduled to receive the second or third telephone call will be randomly selected for the quality checks. Participant details will be cross-checked on the study telephone by the quality control officers at each study site to confirm that the participant received the appropriate telephone call. Call logs will also be checked to confirm the language used in the telephone call vis-à-vis the participant’s preferred language and the correctness of the telephone number that received the call. Research assistants will continue attempting telephone calls if a patient does not answer the telephone, if another person answers, if it is not convenient for a patient to talk, or if the telephone is switched off until an effective call is made to the intended participant. A successful call will be defined as one in which a patient is reached, and a conversation takes place.

#### SMS Reminders and a Transport Incentive

Participants in this arm will receive the same SMS reminder package as those in the SMS reminder–only arm, along with a transport incentive of UGX 10,000 (approximately US $3). This transport incentive will be provided either after submitting a sputum sample in case the participant was unable to produce the sputum on the first day of the health facility visit or upon receiving their test results. The transport incentive will be disbursed through a prepaid mobile money system to ensure accountability for both the patient and the study team and to comply with the ongoing government campaign promoting the use of electronic cash [35]. These interventions will be administered in addition to the SOC provided at the health facility.

#### Telephone Calls and a Transport Incentive

The same telephone call package received by participants in the telephone call–only arm will be offered to those in this arm, along with a transport incentive of UGX 10,000 (approximately US$ 3). This will be in addition to the SOC received at the health facility. This transport incentive will be provided either after submitting a sputum sample in case a participant was unable to produce sputum on the first day of the hospital visit or upon receiving their test results. We will use the prepaid mobile money system to pay the transport incentive to participants in this study arm.

### Sample Size Estimation

An estimated sample size of 2389 participants was determined to achieve 80% power at a type I error of 5%, adjusted for multiple comparisons using the Bonferroni correction. Four comparisons were considered according to the study objectives; therefore, the adjusted level of significance is 1.25%, with a corresponding 2-tailed Z value of 2.238. We also adjusted for a 24% LTFU among patients with TB, based on findings from the formative study that informed the design of these interventions. We assumed a 40% reduction in LTFU based on previous similar studies [36,37] for comparisons between SOC and either SMS reminders alone or telephone calls alone, resulting in an assumed proportion of LTFU of 14.4% in both these groups. For comparisons between SOC and combined interventions (SMS reminders or telephone calls with a transport incentive), a 50% reduction will be considered clinically significant, given the expected higher impact of the incentive. The sample size has been further adjusted for clustering using a design effect of 1.5 to account for the clustering anticipated at the health facility level and a postenrollment attrition rate of 20%.

### Study Population

The study population will comprise persons with presumed TB who did not complete diagnosis on the same day as their health facility visit. Persons with presumed TB will be defined as those presenting with signs and symptoms suggestive of TB, including cough, fever, night sweats, and unexplained weight loss.

We will include (1) patients referred for Xpert MTB/RIF testing; (2) those aged 18 years or older; (3) those currently residing in the health facility’s catchment area; (4) those willing to consent to the study procedures, including follow-ups, and able to sign an informed consent form; (5) those who own a mobile phone and have it in their possession on the day of recruitment; and (6) those able to read (a test SMS text message will be sent to the patient’s mobile phone, and they will be asked to read it aloud). Participants planning to move out of the study area within 3 months after enrollment and those participating in other related studies (eg, those involving SMS reminders, telephone calls, or transport incentives) will be excluded.

### Randomization and Blinding

**Figure 2.**
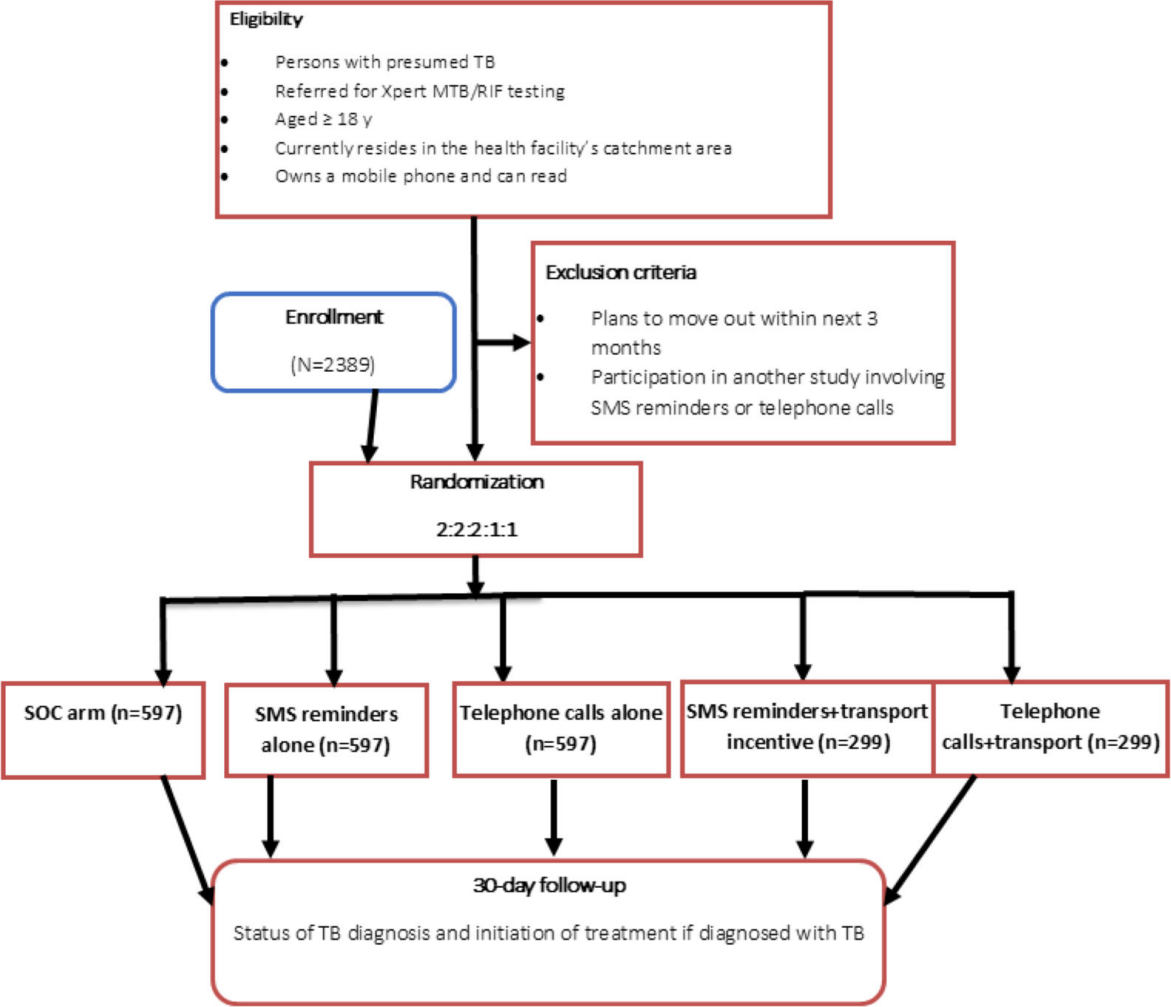
Flow diagram for randomization. SOC: standard of care; TB: tuberculosis.

Eligible participants will be randomly assigned by research assistants to 1 of the 5 study arms using permuted block randomization in a ratio of 2:2:2:1:1 via a predesigned mobile app (Figure 2). The block sizes will be varied to minimize predictability. A computer algorithm will be designed by the biostatistician together with the app designer to generate the allocation sequence in blocks of 8 and 16, assigning their order randomly. The allocation sequence will then be input into the predesigned mobile app for automatic randomization once the research assistants input the participant’s unique study ID number. Due to the nature of the interventions, participants will not be blinded to their assigned study arm. Research assistants will also not be blinded, given their role in delivering the interventions. However, the outcome assessors and data analyst will remain blinded to the study arms.

### Recruitment Strategies for Study Participants

Two research assistants will be strategically positioned at different potential entry points for persons with presumed TB in each study health facility. The entry points will include the outpatient department, antiretroviral therapy (ART) clinic, prenatal clinic, and TB unit. The inpatient units will be excluded because admitted patients are less likely to be lost to follow-up, as they remain within the health facility throughout the testing process. In each study facility, we will hold entry meetings with health workers from the various departments involved in identifying persons with presumed TB to inform them about the study and solicit their support in referring eligible patients throughout the study period.

Referred patients will be introduced to the study team and asked whether they are interested in participating. Those who express interest will be given a card and requested to return to the study desk once they complete care seeking. The card will serve as a reminder not to leave the health facility before returning to the study desk. Patients who return after completing care seeking will be assessed for eligibility, and those who meet the criteria will be asked to provide informed consent by signing the consent form. Eligible participants will then be enrolled and randomly assigned to 1 of the 5 study arms using a smartphone preloaded with the randomization app. All participant details will be recorded in the study registers for persons with presumed TB in both electronic and hard-copy formats for backup.

### Participant Follow-Up Procedures

Follow-up interviews will be conducted after 30 days from the day of recruitment for all participants primarily through face to face interviews at the health facility. Home visits will be conducted for patients who fail to turn up at the health facility. If still we can not access the patient, telephone calls will be made by research assistants to conduct the interview over the phone. At follow-up, data will be collected on whether the TB tests had been performed, along with the date of the TB test results, the date of registration for TB treatment, and the TB registration number. If a participant does not submit a sputum sample, receive test results, or initiate treatment after diagnosis, they will be asked to provide reasons for not undergoing testing, receiving results, or starting treatment.

### Data Collection Procedures

Participants will be enrolled in the study and assigned a unique ID number, which will be used for randomization using the mobile app. Once the participant has been randomly assigned, baseline measurements and tracking information will be collected and recorded using the Open Data Kit (ODK) platform (Get ODK Inc) and directly uploaded to the server. Data will be collected on age, sex, occupation, date when identified as a person with presumed TB, distance to the health facility of enrollment, test method used (ie, sputum smear microscopy or Xpert MTB/RIF), and knowledge and perceptions about the disease and treatment. We will train outcome assessors to objectively conduct interviews and assess outcomes at follow-up. This approach will minimize any biases associated with the team administering the intervention.

### Trial End Points, Outcomes, and Timelines

The primary end point for the trial is completion of the TB diagnosis process within 30 days of being recruited. Completion of TB diagnosis will be defined as submitting a sputum sample for Xpert MTB/RIF testing and receiving test results within 30 days of being identified as a person with presumed TB. The 30-day follow-up period was selected based on previous studies for comparison [25,33]. Completion of TB diagnosis will be measured as the proportion of patients who completed TB diagnosis within 30 days out of all persons with presumed TB recruited into the study.

The secondary outcomes will include (1) TB treatment initiation, which will be defined as starting TB treatment within 30 days of being diagnosed (this will be measured as the proportion of patients diagnosed with TB who initiated treatment out of the total number of patients diagnosed with TB recruited into the study); (2) median turnaround times for TB diagnosis and treatment initiation; and (3) acceptability and feasibility of the study interventions. The detailed procedures and timelines are indicated in Table 1.

**Table 1.** Study procedures and timelines.

Study procedure	Screening	Baseline	Day 0	Results available	Day after results reminder	Day 30
Enrollment						
Eligibility screening	✔					
Informed consent	✔					
Allocation	✔					
Sociodemographic information		✔				
Clinical history		✔				
Knowledge of and attitudes regarding TB^a^		✔				
Interventions						
SMS reminders			✔	✔	✔	
Telephone calls			✔	✔	✔	
Transport incentives				✔		
Assessments						
Sputum submission						✔
Receipt of results						✔
Receipt of interventions						✔
Treatment initiation for those diagnosed						✔
Acceptability assessment						✔
Qualitative study on perceptions and experiences of the interventions						✔

a TB: tuberculosis.

### Data Management and Analysis

#### Overview

ODK data will be downloaded into Microsoft Excel and transferred to Stata 14 (StataCorp LLC) for cleaning and analysis. The primary analytical approach will follow the intention-to-treat principle, with participants in the intervention arm considered exposed to the intervention regardless of whether they received specific intervention services. We will assess for any contamination by conducting and comparing the results of the intention-to-treat and per-protocol analyses. Descriptive statistics (means, medians, and proportions) will be used to describe participant characteristics across the study arms. We will control for characteristics that are not balanced across the study arms in the fitted regression models. Chi-square tests will be performed for categorical outcomes. Log-binomial regression models using generalized linear modeling will be applied to assess associations between each intervention and the outcomes. A *P* value <.05 will be considered statistically significant.

#### Interim Analysis

Although this trial does not pose a significant risk to participants, we plan to conduct an interim analysis. Findings from the interim analysis will be shared with the data and safety monitoring board (DSMB) to determine whether the trial should continue based on the emerging results.

### Ethical Considerations

The study was approved by the Makerere University School of Public Health Research and Ethics Committee (799) and the Uganda National Council of Science and Technology (HS993ES). The study will be conducted in accordance with the World Medical Association Declaration of Helsinki. Written informed consent will be obtained from all participants. Each participant will receive compensation of 10,000 Ugandan shillings (US $3) only for their time in the study at each level of data collection. All hard-copy study materials will be stored securely under lock and key, with access restricted to core project staff. Electronic data will be stored on a password-protected server hosted at Verton IT Ltd, with access similarly restricted to core project staff. To minimize the risk of inadvertent disclosure of presumptive TB status through SMS reminders or telephone calls received by unintended recipients, we will ensure that the SMS reminder content is neutral, with no mention of the word “TB,” serving only as a reminder to the patient to return to the health facility. This approach was adopted based on feedback from the co-design workshop with stakeholders, who recommended avoiding the use of the word “TB” in the SMS reminder content. We will also only recruit participants who personally own a mobile phone and do not share it with others. Furthermore, at the beginning of each telephone call, participants will be asked to identify themselves to confirm that the data collectors are speaking with the correct individual. All enrolled participants will be assigned a unique ID number at recruitment, which will be used throughout the study period. All data will be deidentified before analysis. The trial has been registered at ClinicalTrials.gov (NCT05964842).

### Study Oversight and Monitoring

The study investigators, coordinator, supervisors, and research assistants will meet daily during participant recruitment for quality control purposes. This trial will be monitored by an independent DSMB and a local steering committee (LSC). The DSMB will serve as the core technical team responsible for reviewing cumulative trial data, monitoring adherence to the trial protocol, and ensuring patient safety. It will also review any suggestions for protocol amendments. The DSMB will comprise 3 members (an epidemiologist, a clinical officer, and a senior researcher) and meet every 2 months to assess trial progress. The LSC will provide administrative oversight, ensuring adherence to study procedures, monitoring trial progress, and providing advice and guidance. It will comprise 7 members (a community advisory member, an IT specialist, 2 policymakers, a biostatistician, an epidemiologist, and a social worker) and meet every 6 months.

### Adverse Events Monitoring and Reporting

The planned trial interventions pose minimal risk to participants; hence, we do not anticipate serious adverse events. However, we will continually conduct risk assessments to identify any incidents that may occur. The research assistants will be trained to monitor for adverse events, document them accurately, and follow established reporting procedures. All adverse events occurring during the study, whether or not considered related to the study, will be reported in writing to the study team, the LSC, the DSMB, and the Makerere University School of Public Health Research and Ethics Committee for assessment and further review. Each event will be recorded in the study registry, documenting the following information: description of the event, date of occurrence, participant details, onset and end dates, and severity. We will actively follow up each event until resolution or stabilization.

### Dissemination Plan for Research Findings

All findings from the trial will be disseminated in workshops and conferences as oral or poster presentations. The target audience will include members of the Uganda National TB and Leprosy Program research forum, policymakers, TB program implementing partners, academic researchers, and the study participants. Policy briefs will be prepared, and a report of the study findings will be submitted to the participating health facilities and to the funder, the European and Developing Countries Clinical Trials Partnership. All findings will be published in open-access, peer-reviewed journals.

## Results

The study was funded in June 2020 and data collection for this RCT started in August 2023. We encountered delays during the COVID-19 period hence the RCT started at a later date than anticipated. The results are not yet available. Results from the preliminary analysis will be ready by December 2025. As of August 28, 2025, a total of 2355 participants had been recruited, and preliminary analyses are ongoing. The final results are expected to be published by March 2026.

## Discussion

### Summary

The MILEAGE4TB study is an individual randomized controlled trial to determine the effect of SMS reminders, telephone calls, and transport incentives on enhancing completion of TB diagnosis among persons with presumed TB and initiation of treatment for diagnosed patients. The study hypothesizes that, compared to SOC, each individual intervention—SMS reminders alone, telephone calls alone, or a combined intervention of either SMS reminders or telephone calls with transport incentives—will reduce prediagnosis LTFU. Some of the factors contributing to patient LTFU are high patient loads, cartridge shortages, and low staffing levels in laboratories [31,32]. In addition, evidence indicates that even with improved diagnostic tools such as Xpert MTB/RIF, same-day results are often not feasible in low- and middle-income countries (LMICs) due to the unavailability of Xpert MTB/RIF equipment and thus the need to refer specimens for testing. These delays prevent same-day result delivery, increasing the likelihood of patient LTFU.

This study will address cascading patient LTFU along the TB care continuum, particularly at 2 time points: before diagnosis and after treatment*.* Prediagnosis and pretreatment LTFU are important because untreated patients with TB remain infectious and can continue to transmit TB in the community, while failure to initiate treatment results in poor patient outcomes, including death [6,24,38]. Studies have shown that timely diagnosis and treatment improve patient outcomes [39,40]. Effective TB case detection has been reported to lower TB incidence, prevalence, and mortality levels [41]. Therefore, routine follow-up of patients during the process of diagnosis and linkage to treatment is important. This is in line with the targets of the “End TB Strategy” and the United Nations’ Sustainable Development Goals [42]. In addition, instances of pretreatment LTFU are not included in routine reporting by national TB programs, leading to an overestimation of program effectiveness [43].

The proposed intervention uses SMS reminders and telephone calls to improve communication between patients and health care providers, while transport incentives address the economic barriers associated with completion of TB diagnosis and treatment initiation. Telephone-based interventions such as SMS reminders and telephone calls have successfully improved adherence to TB treatment in Uganda [44-46] and increased the uptake of other health interventions, such as childhood immunization [47]. In addition, incentives have been found to improve health care outcomes [48-50]. However, there is limited data from LMICs on the role of SMS reminders, telephone calls, and transport incentives in interventions seeking to improve completion of TB diagnosis and initiation of treatment for diagnosed patients. This study will provide evidence on the use of mHealth strategies and incentives to improve completion of TB diagnosis and initiation of treatment for diagnosed patients.

Given that the phase of being presumed for TB offers an opportunity for early diagnosis and treatment initiation [51,52], this study, whose aim is to enhance completion of TB diagnosis and initiation of treatment for diagnosed patients, is critical to achieving the targets of the “End TB Strategy” [2]. In addition, our study will put into practice the “Find. Treat. All.” initiative, which seeks to close gaps in TB detection and treatment. This initiative, launched in 2018 by the WHO in collaboration with the Stop TB Partnership and the Global Fund to Fight AIDS and Tuberculosis, focuses on improving the quality of TB services through assessment of the TB care cascade and identification of missing cases [53]. Furthermore, the provision of transport incentives is in line with the principles of social protection for patients and supports the attainment of universal health coverage, as outlined in Sustainable Development Goal 3 and the “End TB Strategy” [42].

### Study Strengths and Limitations

This randomized controlled trial is expected to provide one of the highest levels of evidence for ways to enhance completion of the TB diagnostic process and treatment initiation. The study will be conducted across different levels of health facilities to enhance representativeness and increase the generalizability of the findings. In addition, we are implementing a multifaceted intervention comprising SMS reminders, telephone calls, and transport incentives, which is an approach that, to the best of our knowledge, has not been previously evaluated among persons with presumptive TB.

However, blinding will not be possible at all levels, particularly for participants, which may introduce some bias. This risk will be minimized through continual clarification of the study goal and methods. Moreover, individuals who share mobile phones will be excluded to avoid inadvertent disclosure of their testing status. This exclusion criterion may limit participation of certain populations that could have benefited from this intervention.

### Conclusions

This study will assess the effect of SMS reminders, telephone calls, and transport incentives on enhancing completion of TB diagnosis among persons with presumed TB and treatment initiation for diagnosed patients within Uganda’s health care system. The trial will provide evidence that can inform health care practice and policy related to the TB diagnosis process, which could ultimately improve patient care. The use of SMS reminders and telephone calls in the phase of being presumed for TB will provide insights on their effectiveness in this phase, which has not been extensively explored. Moreover, the application of these technologies will add to existing evidence on the effectiveness of mHealth in improving health service delivery.

## Abbreviations

DSMB: data and safety monitoring board
HC: health center
LMICs: low- and middle-income countries
LSC: local steering committee
LTFU: loss to follow-up
mHealth: mobile health
ODK: Open Data Kit
SOC: standard of care
SPIRIT: Standard Protocol Items: Recommendations for Interventional Trials
TB: tuberculosis
WHO: World Health Organization
